# Unilateral extrapedicular vs. bilateral transpedicular percutaneous kyphoplasty in osteoporotic vertebral compression fractures: an exploratory systematic review and meta-analysis

**DOI:** 10.3389/fsurg.2026.1854774

**Published:** 2026-06-12

**Authors:** Yihao Tao, Jingsong Cheng, Bo Wang, Hekai Shi, Wu Wang, Chao Yin, Sheng Chen

**Affiliations:** 1 Department of Orthopedics, Union Hospital, Tongji Medical College, Huazhong University of Science and Technology, Wuhan, China; 2 Department of Medical Oncology, Sun Yat-sen University Cancer Center, Guangzhou, China; 3 Department of Rehabilitation, Wuhan No. 1 Hospital, Wuhan, China; 4 Department of Vascular Surgery, Fudan University Affiliated Huadong Hospital, Shanghai, China; 5 Department of Orthopedics, Fifth Affiliated Hospital of Xinjiang Medical University, Urumqi, China; 6 Xinjiang Key Laboratory of Natural Medicines Active Components and Drug Release Technology, Engineering Research Center of Xinjiang and Central Asian Medicine Resources, Ministry of Education, College of Pharmacy, Xinjiang Medical University, Urumqi, China

**Keywords:** bilateral transpedicular approach, meta-analysis, osteoporotic vertebral compression fracture, percutaneous kyphoplasty, unilateral extrapedicular approach

## Abstract

**Background:**

Severe osteoporotic vertebral compression fractures (OVCFs) may require uniform and symmetrical cement distribution to achieve adequate biomechanical stability. Evidence for the unilateral extrapedicular approach (UEA) in percutaneous kyphoplasty (PKP) is limited. This study compares the safety and efficacy of UEA vs. bilateral transpedicular approach (BTA) in PKP for OVCFs.

**Methods:**

Prospective and retrospective studies comparing UEA and BTA in PKP were identified through searches of Web of Science, PubMed, Embase, Scopus, and the Cochrane Library. Studies published through January 2026 were included. The study was performed using R version 4.5.1 with the meta package.

**Results:**

Four studies, including 394 OVCF patients, were analyzed. UEA-PKP and BTA-PKP demonstrated comparable efficacy in Oswestry Disability Index score, Visual Analog Scale score, kyphotic correction, and vertebral height restoration. However, relative to BTA-PKP, UEA-PKP results in shorter operative duration (MD = −8.43, 95% CI: −10.90 to −5.96), reduced radiation exposure (MD = −7.23, 95% CI: −11.31 to −3.15), lower cement volume (MD = −0.94, 95% CI: −1.57 to −0.32), and is associated with lower cement leakage rate (OR = 0.49, 95% CI: 0.28–0.85).

**Conclusion:**

UEA-PKP may offer safety advantages while maintaining similar efficacy compared with BTA-PKP. However, these findings should be interpreted as exploratory, as they are based exclusively on a limited number of retrospective cohort studies.

## Introduction

Osteoporotic vertebral compression fractures (OVCFs) represent one of the most common clinical consequences of osteoporosis, particularly in older individuals. OVCFs often result in persistent back pain, progressive spinal deformity, and substantial reduction in quality of life (1, 2). As the global population ages, the incidence of OVCFs is increasing, posing significant clinical, economic, and societal burdens worldwide (3, 4).

**Figure 1:**
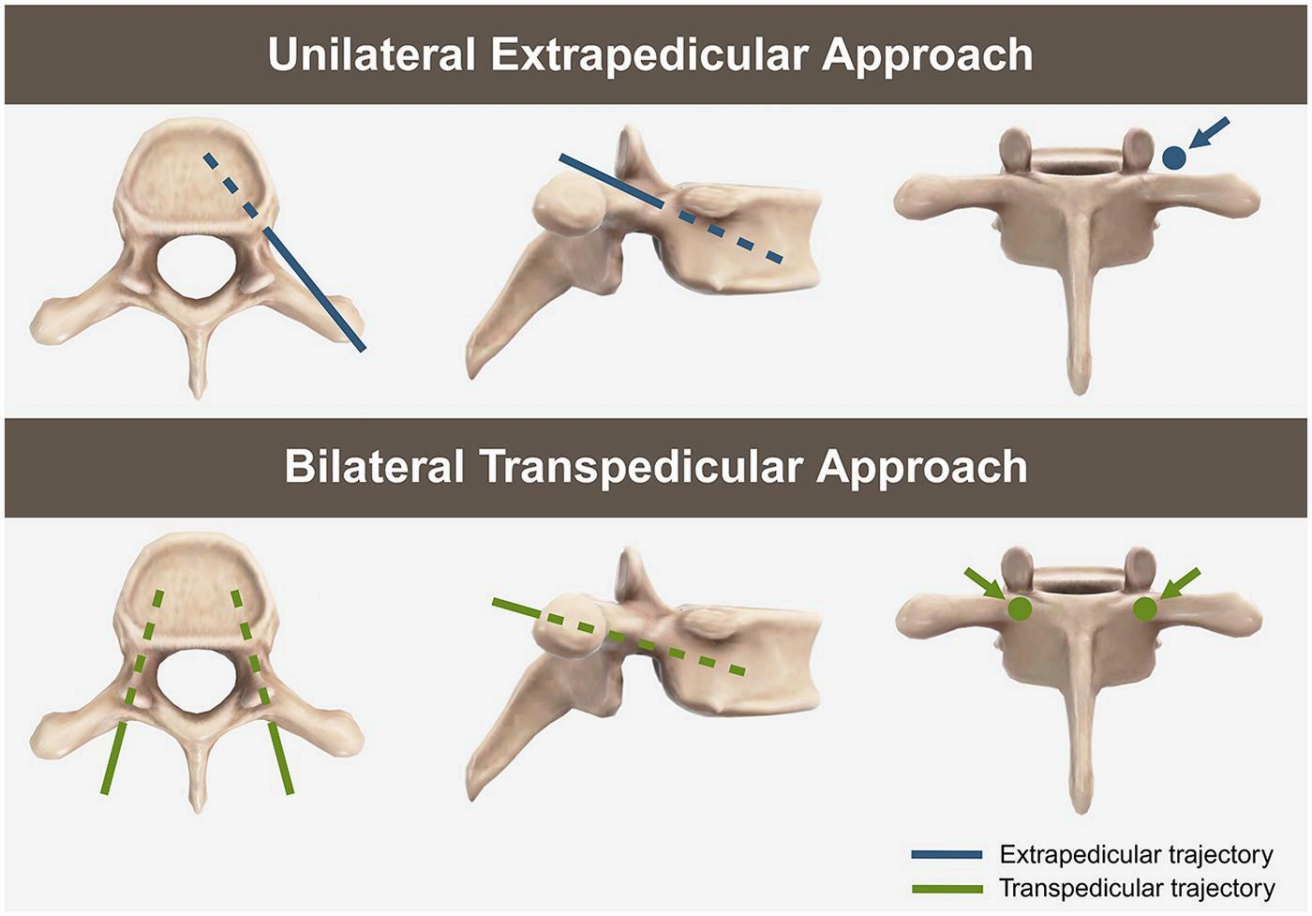
The puncture paths of UEA and BTA in PKP.

Percutaneous kyphoplasty (PKP) is widely employed in the treatment of OVCFs, as it is minimally invasive, facilitates vertebral height restoration, and improves patients' quality of life (5). The unilateral transpedicular approach (UTA) is more commonly adopted, as it results in shorter operative duration, reduced radiation exposure, and lower treatment cost (6–8). For patients with severe OVCFs, particularly those with central or bilateral vertebral collapse, uniform and symmetrical cement distribution may often be required to achieve adequate biomechanical stability (9, 10). However, because UTA is performed through a transpedicular route, the limited abduction angle hinders uniform and symmetrical cement distribution, potentially increasing the long-term risk of spinal imbalance and vertebral refracture (11). Therefore, the bilateral transpedicular approach (BTA) is often adopted in these patients. Recently, the unilateral extrapedicular approach (UEA) has emerged, enabling uniform cement distribution by advancing the needle at a wider abduction angle toward the vertebral midline. Importantly, UEA preserves the advantages of a unilateral approach (Figure 1) (12, 13). Nevertheless, evidence regarding the efficacy and safety of UEA remains limited and inconsistent.

Although several meta-analyses have compared the unilateral and bilateral approaches in PKP, these studies were restricted to the transpedicular approach (6–8). No meta-analysis has systematically evaluated the safety and efficacy of UEA-PKP in comparison with BTA-PKP. Although the potential advantages of UEA-PKP may be particularly relevant in severe OVCFs, most currently available studies included patients with varying fracture severities. Nevertheless, this meta-analysis still provides valuable evidence regarding the comparative safety and efficacy of UEA-PKP and BTA-PKP.

## Materials and methods

### Protocol and registration

This study was conducted in accordance with the updated PRISMA guidelines (14) with prior registration in the PROSPERO database (CRD42023492950).

### Information sources and search strategy

A systematic literature search was performed across PubMed, Embase, Web of Science, Scopus, and the Cochrane Library from database inception to January 2, 2026. The search terms consisted of “percutaneous kyphoplasty,” “osteoporotic vertebral compression fracture,” “transpedicular,” “extrapedicular,” “modified,” “OVCF,” “PKP,” and “Kummell” (see Supplementary Table S1 for details). Additionally, printed materials and institutional archives at the Huazhong University of Science and Technology Library were manually screened with assistance from librarians. All searches were conducted independently by two reviewers following a prespecified protocol, with disagreements resolved through consultation with a third reviewer. In addition, reference lists of eligible studies and relevant reviews were examined to identify additional eligible studies.

### Eligibility criteria

Studies were included when they satisfied the following criteria:

1. Study design: Eligible designs included randomized controlled trials, cohort studies, and case–control studies.
2. Participants: Adult patients with radiologically confirmed OVCF (x-ray, CT, or MRI), including both single-level and multiple-level fractures.
3. Interventions: Comparison between UEA-PKP and BTA-PKP.
4. Outcomes: Operative duration, fluoroscopy times (number of fluoroscopic exposures), cement leakage rate, injected cement volume, Visual Analog Scale (VAS) (15), Oswestry Disability Index (ODI) (16), vertebral body height, and Cobb/kyphotic angle.

### Data extraction and quality assessment

Data were extracted independently by two reviewers using a standardized form, and discrepancies were resolved through discussion with a third reviewer. Extracted information included the first author, study design, sample size, sex distribution, patient age, follow-up duration, fracture levels (thoracic vs. lumbar), baseline VAS/ODI scores, and outcome measures. Outcomes were categorized as safety outcomes (operative duration, fluoroscopy time, cement leakage rate, and injected cement volume) and efficacy outcomes (VAS, ODI, vertebral body height, and Cobb or kyphotic angle).

The quality of the included cohort studies was evaluated using the Newcastle-Ottawa Scale (NOS) (17). The NOS assesses study quality across three key domains: selection of participants, comparability of groups, and exposure/outcome assessment, with a maximum score of nine points. Higher scores reflect greater methodological quality, and studies scoring ≥6 points were classified as high-quality.

### Statistical analysis

All statistical analyses were performed using R software (version 4.5.1) with the meta package. Dichotomous outcomes were summarized as odds ratios (ORs) with corresponding 95% confidence intervals (CIs), whereas continuous outcomes were synthesized as mean differences (MDs) or standardized mean differences (SMDs) with 95% CIs (18). Cobb angle and kyphotic angle were combined using SMDs for meta-analysis.

Between-study heterogeneity was assessed using the Chi-square test and the *I*^2^ statistic. A fixed-effects model was applied when heterogeneity was low (*P* > 0.05 and *I*^2^ < 50%); otherwise, a random-effects model was used (19, 20). Subgroup and sensitivity analyses were performed to investigate potential sources of heterogeneity. Sensitivity analysis was performed using a leave-one-out approach, sequentially omitting each study to examine the robustness of the pooled estimates. Publication bias was evaluated using funnel plots, with the trim-and-fill method applied to adjust for potential asymmetry. All statistical tests were two-sided, with *P* values < 0.05 considered statistically significant.

## Results

### Literature search

**Figure 2:**
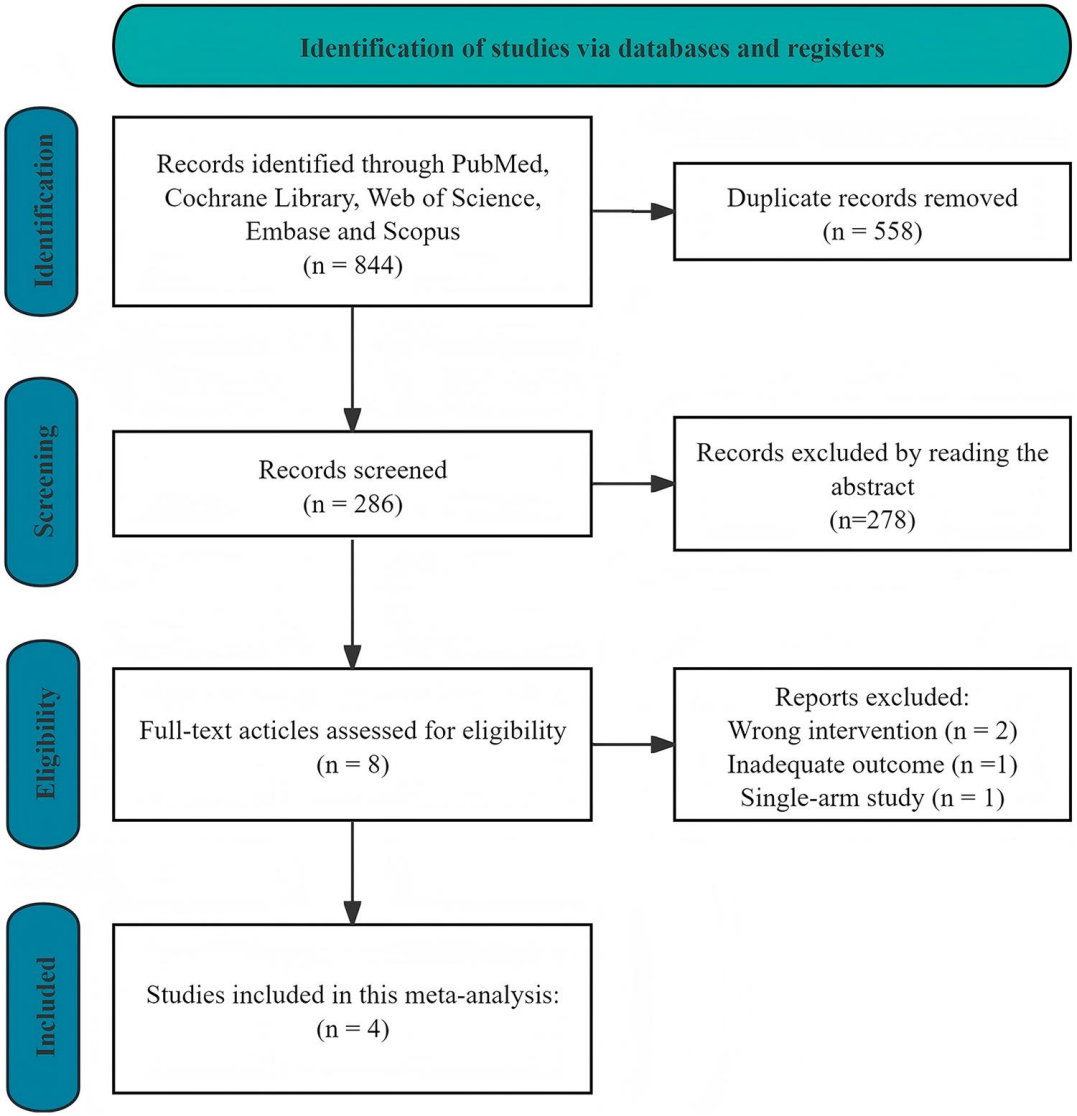
Flowchart of study selection for meta-analysis.

The database search yielded 844 records. After removal of 558 duplicate records, 286 records underwent title and abstract screening, and 278 records were subsequently excluded. Full-text review was performed for 8 potentially relevant studies, among which 4 met the inclusion criteria and were included in the final analysis. The study selection workflow is illustrated in Figure 2.

### Characteristics and quality assessment of included studies

Four retrospective cohort studies (21–24) were eligible for inclusion, comprising 394 patients, including 187 treated with UEA-PKP and 207 treated with BTA-PKP. No prospective studies met the inclusion criteria. All included studies enrolled patients with single-level OVCFs. These studies were published from 2021 to 2024 and all reported follow-up durations of at least 12 months. Methodological quality was evaluated using the NOS. Two studies scored 8 points (21, 22), and two studies scored 9 points (23, 24), suggesting generally high methodological quality (see Supplementary Table S2 for detailed scoring). Baseline characteristics of the included studies and their corresponding NOS scores are presented in Table 1.

**Table 1:** Baseline characteristics of the included studies.

First author, year	Design	Group	Cases	Sex (Male/Female)	Age (years)	Follow-up (months)	Thoracic levels	Lumbar levels	Baseline VAS	Baseline ODI	Quality assessment
He 2022 (21)	Cohort	UEA	47	-	>65	12	17	30	7.06 ± 1.17	70.32 ± 6.64	NOS: 8
		BTA	42	-	>65	12	18	24	6.67 ± 0.75	68.69 ± 4.75	
Xu 2024 (23)	Cohort	UEA	62	25/37	69.4 ± 5.2	12	26	36	7.0 ± 0.7	70.7 ± 4.3	NOS: 9
		BTA	74	32/42	68.8 ± 7.8	12	35	39	6.8 ± 0.8	71.5 ± 5.4	
Zhu 2021 (24)	Cohort	UEA	34	5/29	70.1 ± 8.7	16.6 (12–26)	32	2	7.9 ± 0.9	73.7 ± 8.5	NOS: 9
		BTA	42	8/34	71.4 ± 6.8	16.6 (12–26)	39	3	7.7 ± 0.9	73.0 ± 9.0	
Pan 2021 (22)	Cohort	UEA	44	16/28	68.8 ± 5.0	15.3 (12–20)	0	44	7.1 ± 0.8	78.1 ± 4.4	NOS: 8
		BTA	49	20/29	67.5 ± 4.9	15.3 (12–20)	0	49	7.1 ± 1.0	79.7 ± 4.9	

UEA, unilateral extrapedicular approach; BTA, bilateral transpedicular approach; VAS, Visual Analog Scale; ODI, Oswestry Disability Index; NOS, Newcastle-Ottawa Scale.

### Operative duration

**Figure 3:**
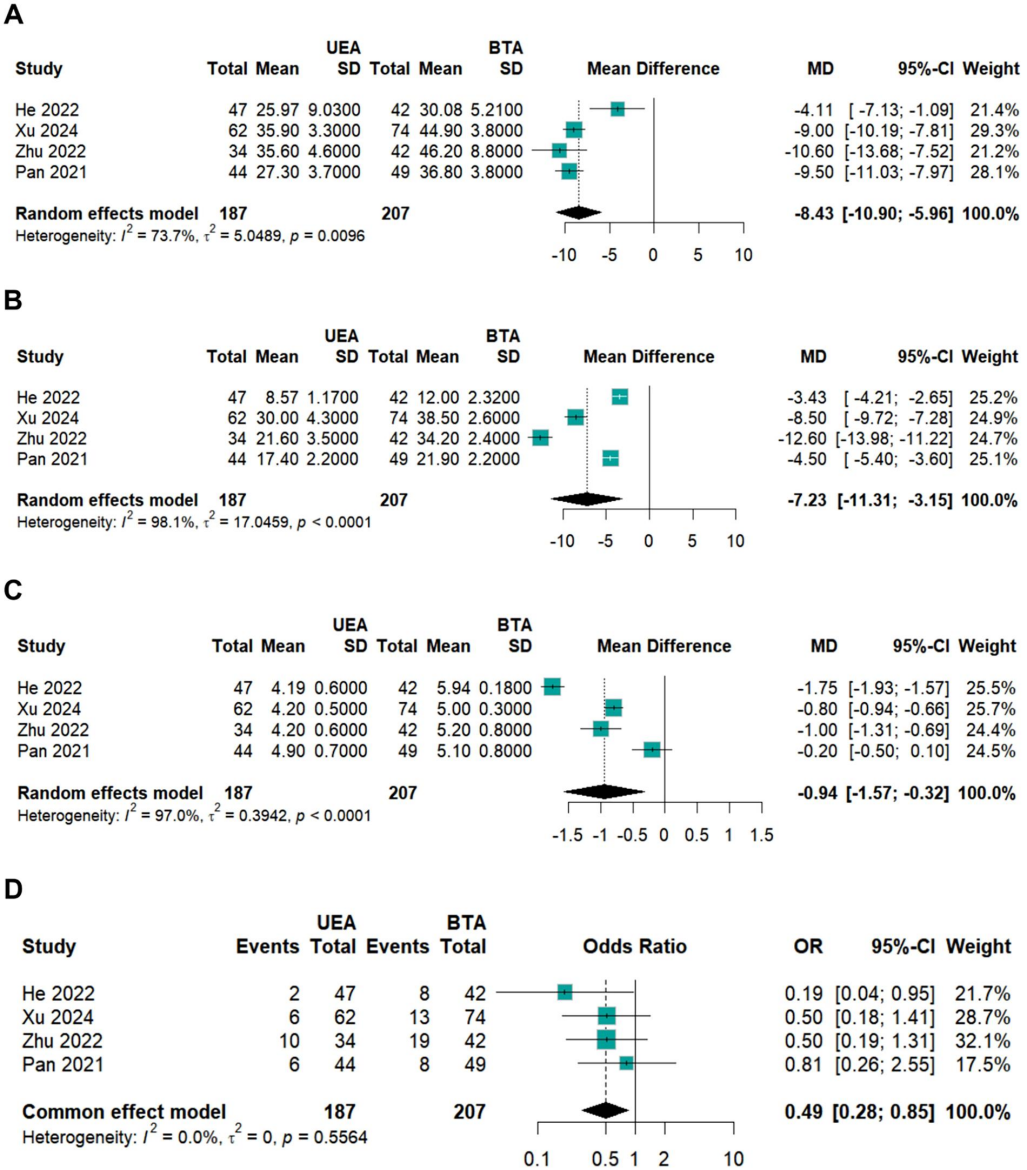
Forest plots of safety outcomes comparing UEA-PKP and BTA-PKP: **(A)** operative duration, **(B)** fluoroscopy times, **(C)** injected cement volume, **(D)** cement leakage rate.

Four studies provided operative duration (21–24). The results showed high heterogeneity (*P* = 0.0096, *I*^2^ = 73.7%) across the studies, and a random effects model was used. The operative duration was significantly shorter in the UEA group than in the BTA group (MD = −8.43, 95% CI: −10.90 to −5.96) (Figure 3A). Despite the substantial heterogeneity, all included studies consistently favored shorter operative duration in the UEA group, although the effect magnitude varied across studies.

### Fluoroscopy times

Four studies reported fluoroscopy times (21–24). Heterogeneity across the studies was high (*P* < 0.0001, *I*^2^ = 98.1%). Therefore, a random effects model was used. The fluoroscopy times of the UEA group were significantly fewer than those of the BTA group (MD = −7.23, 95% CI: −11.31 to −3.15) (Figure 3B). Although all included studies consistently favored fewer fluoroscopy times in the UEA group, substantial heterogeneity was observed, and the findings should therefore be interpreted with caution.

### Injected cement volume

Four studies reported injected cement volume (21–24). The results showed high heterogeneity (*P* < 0.0001, *I*^2^ = 97.0%) across the studies. Therefore, a random effects model was used. The UEA group had a lower cement volume than the BTA group (MD = −0.94, 95% CI: −1.57 to −0.32) (Figure 3C). Although all included studies consistently favored lower cement volume in the UEA group, substantial heterogeneity was observed, and the findings should therefore be interpreted with caution.

### Cement leakage rate

Cement leakage rate was available from 4 studies (21–24). The results showed no heterogeneity (*P* = 0.5564, *I*^2^ = 0.0%) across the studies. A fixed effects model was used. The cement leakage rate was significantly lower in the UEA group than in the BTA group (OR = 0.49, 95% CI: 0.28–0.85) (Figure 3D).

### VAS

**Figure 4:**
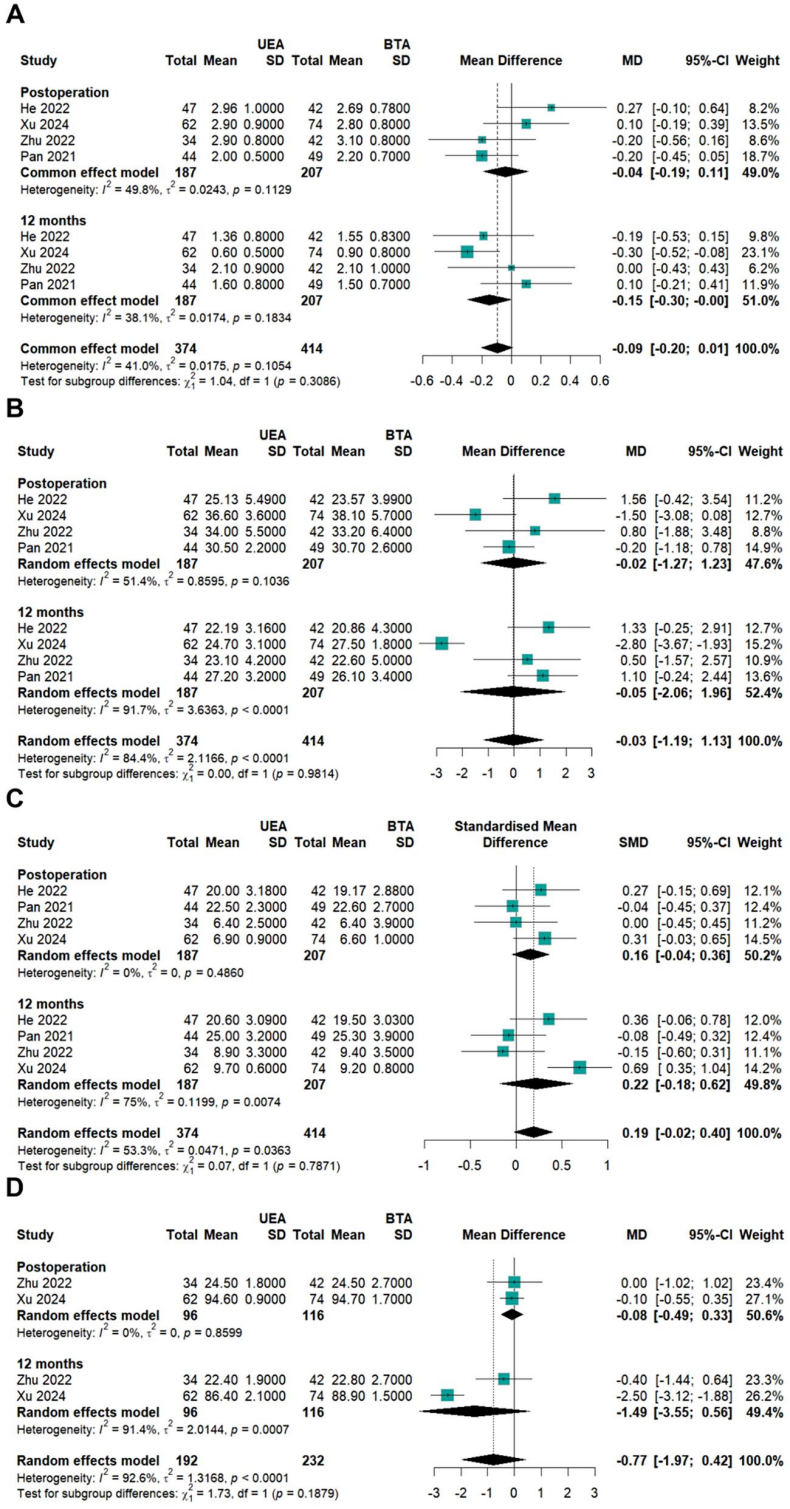
Forest plots of efficacy outcomes comparing UEA-PKP and BTA-PKP: **(A)** VAS, **(B)** ODI, **(C)** Cobb/kyphotic angle, **(D)** vertebral body height.

VAS was available from 4 studies (21–24). The results showed low heterogeneity (*P* = 0.1054, *I*^2^ = 41.0%) across the studies, and a fixed effects model was employed. In subgroup analysis, the two groups showed comparable VAS at postoperative (MD = −0.04, 95% CI: −0.19–0.11) and 12 months (MD = −0.15, 95% CI: −0.30–0.00). There was low heterogeneity among the subgroups (*P* = 0.3086). There was no significant difference between the two groups when all results were combined (MD = −0.09, 95% CI: −0.20–0.01) (Figure 4A).

### ODI

ODI was provided by 4 studies (21–24). Heterogeneity across studies was high (*P* < 0.0001, *I*^2^ = 84.4%), and a random effects model was employed. In subgroup analysis, the two groups showed comparable ODI at postoperative (MD = −0.02, 95% CI: −1.27–1.23) and 12 months (MD = −0.05, 95% CI: −2.06–1.96). There was low heterogeneity among the subgroups (*P* = 0.9814). The overall outcomes of the ODI revealed no significant difference between the two groups (MD = −0.03, 95% CI: −1.19–1.13) (Figure 4B).

### Cobb/kyphotic angle

Four studies reported Cobb angle or kyphotic angle (21–24). The results showed high heterogeneity (*P* = 0.0363, *I*^2^ = 53.3%) across the studies. Therefore, a random effects model was used. In subgroup analysis, the two groups showed comparable Cobb/kyphotic angle at postoperative (SMD = 0.16, 95% CI: −0.04–0.36) and 12 months (SMD = 0.22, 95% CI: −0.18–0.62), with no heterogeneity among the subgroups (*P* = 0.7871). There was no significant difference between the two groups when all results were combined (SMD = 0.19, 95% CI: −0.020.40) (Figure 4C).

### Vertebral body height

Two studies reported vertebral body height (23, 24). Heterogeneity across studies was high (*P* < 0.0001, *I*^2^ = 92.6%), and a random effects model was employed. In the subgroup analysis, no significant difference was observed between the two groups at postoperative (MD = −0.08, 95% CI: −0.49–0.33) and 12 months (MD = −1.49, 95% CI: −3.55–0.56). There was low heterogeneity among the subgroups (*P* = 0.1879). Based on limited data, no significant difference in vertebral body height was detected between the two groups (MD = −0.77, 95% CI: −1.97–0.42) (Figure 4D).

### Sensitivity analysis and publication bias

Sensitivity analyses demonstrated that the majority of outcomes were stable. The outcomes of operative duration, fluoroscopy times, cement volume, VAS, and ODI were not affected by the exclusion of any single study, supporting the robustness of these findings. The findings regarding cement leakage rate and Cobb/kyphotic angle were not robust and should therefore be interpreted with caution. For cement leakage rate, the pooled effect became non-significant after omitting one study (21). For Cobb/kyphotic angle, the overall direction of effect was preserved; however, statistical significance emerged after excluding either of the two individual studies (23, 24). The instability of some results is likely attributable to the limited number of available studies (see Supplementary Table S3 for details).

Publication bias was evaluated based on a funnel plot for the primary outcome (cement leakage rate), which showed no apparent asymmetry. The trim-and-fill analysis did not identify any potentially missing studies, and the pooled effect size remained stable after adjustment. Overall, no clear evidence of publication bias was detected. However, these findings should be interpreted cautiously due to the limited number of included studies (Supplementary Figure S1).

## Discussion

BTA is commonly employed in OVCFs requiring substantial fracture reduction to achieve more uniform bone cement distribution (25). Nevertheless, BTA entails increased operative time, treatment cost, and radiation exposure (6–8). UEA can also achieve uniform cement distribution by advancing the needle at a wider abduction angle toward the vertebral midline (13). Importantly, UEA preserves the advantages of a unilateral approach. However, evidence regarding the efficacy and safety of UEA remains limited, and the lack of a standardized puncture protocol may restrict its broader clinical adoption.

Compared with BTA, UEA results in shorter operative duration and reduced radiation exposure. UEA retains the inherent advantages of a unilateral technique. Moreover, the extrapedicular route enables flexible adjustment of needle trajectory within the paravertebral soft tissues, unconstrained by the vertebral pedicle (26). This flexibility allows the needle to reach the target site efficiently, reducing the need for repeated fluoroscopic guidance and potentially decreasing radiation exposure to both patients and surgeons (9). Many OVCF patients are elderly with cardiopulmonary comorbidities and limited tolerance for prolonged prone procedures (27). Therefore, UEA represents a minimally invasive and effective therapeutic option for this patient population.

The volume of bone cement required for UEA is lower than that used in BTA. Excessive cement may adversely affect neurological and reproductive function (28) and is a risk factor for vertebral refracture and adjacent vertebral fractures postoperatively (29). Compared with BTA, UEA is associated with a lower incidence of cement leakage. Several factors may account for this difference: (1) Optimal cement distribution is generally considered to occupy the anterior two-thirds of the vertebral body (30). In UEA, cement mainly occupies the anterior and middle of the vertebral body (31, 32). Due to the limited puncture angle, cement in the transpedicular approach often spreads laterally and posteriorly within the vertebral body (33). (2) Larger cement volumes increase the risk of leakage (34), and UEA typically requires less cement than BTA. (3) UEA preserves the integrity of the pedicle cortex, thereby lowering the risk of cement leakage caused by pedicle fractures. Nevertheless, the extrapedicular approach poses a risk of paravertebral cement leakage at the vertebral entry point, which may cause thermal injury to the lumbar nerve roots (24).

VAS and ODI scores were similar between the two groups, suggesting comparable levels of pain relief and functional recovery. Both approaches also demonstrated similar effectiveness in correcting kyphotic deformity and restoring vertebral body height. Zhu et al. reported that adequate and well-distributed bone cement can relieve pain and improve functional outcomes, independent of the puncture technique used (24). However, BTA poses a potential risk of facet joint invasion, which may contribute to residual postoperative back pain (35, 36), whereas UEA may result in segmental artery injury during lumbar spine treatment (37, 38). Zhu et al. recommended that, when employing UEA, the puncture trajectory should pass through the bottom of the Kambin triangle, avoiding nerves and arteries (24).

Substantial heterogeneity was observed in several pooled outcomes. This variability may be attributable to differences in surgical techniques, patient characteristics, and outcome measurements across the included studies, which may limit the precision of the pooled estimates. Due to the limited number of included studies and insufficient stratified data, further meta-regression or subgroup analyses may not have been feasible.

This meta-analysis has several limitations. First, only four retrospective cohort studies were included. Although these studies were evaluated as high-quality using the NOS, this tool provides a global quality score but does not systematically evaluate confounding or selection bias. Second, although the clinical background focuses on severe OVCFs that may require bilateral cement distribution, most included studies involved patients with varying fracture severities. Nevertheless, the available data still offer valuable insights for severe cases. Future studies should adopt standardized severity classification systems, such as the Genant classification, to improve clinical interpretation. Third, data regarding recurrent fracture, adjacent-level fracture, and re-operation rates were limited across the included studies, which restricted quantitative synthesis of these outcomes and constrained evidence-based decisions regarding long-term safety. Finally, all included studies originated from Asian populations. Anatomical differences such as pedicle size and extrapedicular safety margins may vary among different populations, which may limit the generalizability of these findings to other populations.

## Conclusion

This study systematically evaluated the safety and efficacy of UEA-PKP compared with BTA-PKP. Both approaches demonstrated comparable efficacy in pain relief, functional improvement, kyphotic correction, and vertebral height restoration. However, relative to BTA-PKP, UEA-PKP results in shorter operative duration, reduced radiation exposure, lower cement volume, and is associated with lower cement leakage rate. Overall, UEA-PKP may offer safety advantages while maintaining similar efficacy. Given that all included studies were a limited number of retrospective cohort studies, the overall level of evidence remains limited, and the findings should be interpreted as exploratory. Further well-designed, multicenter RCTs with larger sample sizes are required before definitive recommendations can be made.

## References

[1] O'Hare B Santaguida P Azizudin AM Papaioannou A. The role of physical performance measures in the physiotherapy assessment and management of older adults with osteoporotic vertebral fractures: a narrative review. Osteoporos Int. (2025) 36:1509–20. 10.1007/s00198-025-07606-x 40643675

[2] Marino V Mungalpara N Amirouche F. Re-evaluating vertebral height restoration assessment in osteoporotic compression fractures: a systematic review and meta-analysis. Eur Spine J. (2025) 34:1641–62. 10.1007/s00586-025-08707-1 39928136

[3] Zhao Y Wang S Feng G. Insights into ChatGPT and DeepSeek application in osteoporotic vertebral compression fractures. Int J Surg. (2025) 111:4955–8. 10.1097/JS9.0000000000002512 40387695

[4] Cho CH Hwang SW Mazanec DJ O'Toole JE Watters WC Annaswamy TM. Guideline summary review: an evidence-based clinical guideline for the diagnosis and treatment of adults with osteoporotic vertebral compression fractures. Spine J. (2025) 25:1670–87. 10.1016/j.spinee.2025.01.016 39894268

[5] Xiao C Wang H Lei Y Xie M Li S. Percutaneous kyphoplasty combined with pediculoplasty for the surgical treatment of osteoporotic thoracolumbar burst fractures. J Orthop Surg Res. (2024) 19:87. 10.1186/s13018-024-04562-w 38254114 PMC10804617

[6] Zhang J Zhou Q Zhang Z Liu G. Comparison between unilateral and bilateral percutaneous kyphoplasty in the treatment of osteoporotic vertebral compression fracture: a meta-analysis and systematic review. Exp Ther Med. (2023) 26:553. 10.3892/etm.2023.12252 37941587 PMC10628642

[7] Cao D-H Gu W-B Zhao H-Y Hu J-L Yuan H-F. Advantages of unilateral percutaneous kyphoplasty for osteoporotic vertebral compression fractures-a systematic review and meta-analysis. Arch Osteoporos. (2024) 19:38. 10.1007/s11657-024-01400-8 38750277

[8] Cheng X Long H-Q Xu J-H Huang Y-L Li F-B. Comparison of unilateral versus bilateral percutaneous kyphoplasty for the treatment of patients with osteoporosis vertebral compression fracture (OVCF): a systematic review and meta-analysis. Eur Spine J. (2016) 25:3439–49. 10.1007/s00586-016-4395-6 26814475

[9] Tan B Yang Q-Y Fan B Lei C Hu Z-M. Is it necessary to approach the severe osteoporotic vertebral biconcave-shaped fracture bilaterally during the process of PKP? J Pain Res. (2021) 14:1601–10. 10.2147/JPR.S293528 34113167 PMC8187090

[10] Lu J Huang L Chen W Luo Z Yang H Liu T. Bilateral percutaneous kyphoplasty achieves more satisfactory outcomes compared to unilateral percutaneous kyphoplasty in osteoporotic vertebral compression fractures: a comprehensive comparative study. J Back Musculoskelet Rehabil. (2023) 36:97–105. 10.3233/BMR-210225 35938239

[11] Tang J Guo W-C Hu J-F Yu L. Unilateral and bilateral percutaneous kyphoplasty for thoracolumbar osteoporotic compression fractures. J Coll Physicians Surg Pak. (2019) 29:946–50. 10.29271/jcpsp.2019.10.946 31564267

[12] Zhu D Hu J-N Wang L Cui W Zhu J-C Ma S. A modified unilateral extrapedicular approach applied to percutaneous kyphoplasty to treat lumbar osteoporotic vertebral compression fracture: a retrospective analysis. Pain Physician. (2023) 26:E191–201. 37192242

[13] Liu L Cheng S Wang Q Liang Q Liang Y Jin W. An anatomical study on lumbar arteries related to the extrapedicular approach applied during lumbar PVP (PKP). PLoS One. (2019) 14:e0213164. 10.1371/journal.pone.0213164 30835754 PMC6400376

[14] Page MJ McKenzie JE Bossuyt PM Boutron I Hoffmann TC Mulrow CD. The PRISMA 2020 statement: an updated guideline for reporting systematic reviews. Br Med J. (2021) 372:n71. 10.1136/bmj.n71 33782057 PMC8005924

[15] Huskisson EC. Measurement of pain. Lancet. (1974) 2:1127–31. 10.1016/s0140-6736(74)90884-8 4139420

[16] Fairbank JC Pynsent PB. The oswestry disability index. Spine. (2000) 25:2940–52. discussion 2952. 10.1097/00007632-200011150-00017 11074683

[17] Stang A. Critical evaluation of the Newcastle-Ottawa scale for the assessment of the quality of nonrandomized studies in meta-analyses. Eur J Epidemiol. (2010) 25:603–5. 10.1007/s10654-010-9491-z 20652370

[18] Lee YH. An overview of meta-analysis for clinicians. Korean J Intern Med. (2018) 33:277–83. 10.3904/kjim.2016.195 29277096 PMC5840596

[19] Maitra S. Fixed-effect versus random-effect model in meta-analysis: how to decide? Indian J Anaesth. (2025) 69:143–6. 10.4103/ija.ija_1203_24 40046706 PMC11878354

[20] Higgins JPT Thompson SG Deeks JJ Altman DG. Measuring inconsistency in meta-analyses. Br Med J. (2003) 327:557–60. 10.1136/bmj.327.7414.557 12958120 PMC192859

[21] He H Tan Y Yang S Zhang C Luo X. Study of unilateral extrapedicular and bilateral pedicle approach percutaneous kyphoplasty for osteoporotic vertebral compression fracture. J Coll Physicians Surg Pak. (2022) 32:924–7. 10.29271/jcpsp.2022.07.924 35795945

[22] Pan H Ding S Zhao X Lin Z Ye H Fu C. Bilateral percutaneous balloon kyphoplasty through unilateral transverse process-extrapedicular approach for osteoporotic vertebral compression fracture of lumbar. Chin J Reparative Reconstr Surg. (2021) 35:1007–13. 10.7507/1002-1892.202103028 PMC8404006 34387430

[23] Xu D Ruan C Wang Y Hu X Ma W. Comparison of the clinical effect of unilateral transverse process extrapedicular and bilateral transpedicular percutaneous kyphoplasty for thoracolumbar osteoporotic vertebral compression fracture. Front Surg. (2024) 11:1395289. 10.3389/fsurg.2024.1395289 39092152 PMC11291213

[24] Zhu D Hu J Wang L Zhu J Ma S Liu B. A comparison between modified unilateral extrapedicular and bilateral transpedicular percutaneous kyphoplasty in the treatment of lumbar osteoporotic vertebral compression fracture. World Neurosurg. (2022) 166:e99–108. 10.1016/j.wneu.2022.06.115 35779757

[25] Chang S Wang Y Cheng S Wang Y-B Liu Y Guo J-W. Assessment of bilateral pedicle root puncture vertebral fissure expansion percutaneous kyphoplasty for the treatment of Kümmell disease. Clin Spine Surg. (2025) 39:E112–9. 10.1097/BSD.0000000000001813 40265711

[26] Zhang H Zhao B Luo L Li P Zhao C Jiang D. The puncture methods of extrapedicular PVP (PKP): a narrative review. Interdiscip Neurosurg. (2021) 25:101250. 10.1016/j.inat.2021.101250

[27] Daher M Sebaaly A Sakr I Daniels AH Schoenfeld AJ. Diagnosis and management of osteoporotic vertebral compression fractures. J Bone Joint Surg Am. (2025) 108:345–54. 10.2106/JBJS.25.00201 41460925

[28] Kakazu C Lippmann M Karnwal A. Theatre team contracts multiple syndromes as a result of bone cement. Br J Anaesth. (2016) 116:303. 10.1093/bja/aev469 26787808

[29] Wang M Li B Wang Y Jiang S Wen G Jiang L. The effects of bone cement volume in percutaneous vertebroplasty for thoracolumbar junction vertebral compression fractures: a clinical comparative study. Mediators Inflamm. (2022) 2022:4230065. 10.1155/2022/4230065 35909661 PMC9337957

[30] Zhou X Meng X Zhu H Zhu Y Yuan W. Early versus late percutaneous kyphoplasty for treating osteoporotic vertebral compression fracture: a retrospective study. Clin Neurol Neurosurg. (2019) 180:101–5. 10.1016/j.clineuro.2019.03.029 30953973

[31] Yan L He B Guo H Liu T Hao D. The prospective self-controlled study of unilateral transverse process-pedicle and bilateral puncture techniques in percutaneous kyphoplasty. Osteoporos Int. (2016) 27:1849–55. 10.1007/s00198-015-3430-5 26608054

[32] Yan L Jiang R He B Liu T Hao D. A comparison between unilateral transverse process-pedicle and bilateral puncture techniques in percutaneous kyphoplasty. Spine. (2014) 39:B19–26. 10.1097/BRS.0000000000000493 25504098

[33] Lien S-B Liou N-H Wu S-S. Analysis of anatomic morphometry of the pedicles and the safe zone for through-pedicle procedures in the thoracic and lumbar spine. Eur Spine J. (2007) 16:1215–22. 10.1007/s00586-006-0245-2 17180401 PMC2200778

[34] Lin D Hao J Li L Wang L Zhang H Zou W. Effect of bone cement volume fraction on adjacent vertebral fractures after unilateral percutaneous kyphoplasty. Clin Spine Surg. (2017) 30:E270–5. 10.1097/BSD.0000000000000204 28323711

[35] Cho S-M Nam Y-S Cho B-M Lee S-Y Oh S-M Kim M-K. Unilateral extrapedicular vertebroplasty and kyphoplasty in lumbar compression fractures: technique, anatomy and preliminary results. J Korean Neurosurg Soc. (2011) 49:273–7. 10.3340/jkns.2011.49.5.273 21716899 PMC3115147

[36] Shen L Yang H Zhou F Jiang T Jiang Z. Risk factors of short-term residual low back pain after PKP for the first thoracolumbar osteoporotic vertebral compression fracture. J Orthop Surg Res. (2024) 19:792. 10.1186/s13018-024-05295-6 39587591 PMC11590304

[37] Ge J Cheng X Li P Yang H Zou J. The clinical effect of kyphoplasty using the extrapedicular approach in the treatment of thoracic osteoporotic vertebral compression fracture. World Neurosurg. (2019) 131:e284–9. 10.1016/j.wneu.2019.07.133 31351209

[38] Ringer AJ Bhamidipaty SV. Percutaneous access to the vertebral bodies: a video and fluoroscopic overview of access techniques for trans-, extra-, and infrapedicular approaches. World Neurosurg. (2013) 80:428–35. 10.1016/j.wneu.2012.09.005 23010067

