# Supplementary material for: Unilateral extrapedicular vs. bilateral transpedicular percutaneous kyphoplasty in osteoporotic vertebral compression fractures: an exploratory systematic review and meta-analysis
Source: Front Surg. 2026 Jun 12;13:1854774. doi: 10.3389/fsurg.2026.1854774 (PMC13299092; doi:10.3389/fsurg.2026.1854774)
Supplement: Supplementary file 1 [file Datasheet1.pdf]

Number	Terms and strategies	Logic
#1	Osteoporotic vertebral compression fracture	(Mesh)
#2	Osteoporotic vertebral compression fracture	(Title/Abstract)
#3	OVCF	(Title/Abstract)
#4	Kummell	(Title/Abstract)
#5	#1 OR #2 OR #3 OR #4	
#6	percutaneous kyphoplasty	(Title/Abstract)
#7	PKP	(Title/Abstract)
#8	#6 OR #7	
#9	transpedicular	(Title/Abstract)
#10	modified	(Title/Abstract)
#11	extrapedicular	(Title/Abstract)
#12	#9 OR #10 OR #11	
#13	#5 AND #8 AND #12	

Supplementary Table S1: Search strategy in online databases.

Study	Selection (score)				Comparability (score)	Outcome (score)			Total score
	Representativeness of the exposed cohort	Selection of the nonexposed cohort	Ascertainment of exposure	Outcome of interest was not present at start of study	Based on the design or analysis	Assessment of outcome	Follow-up long enough for outcomes to occur	Adequacy of follow-up of cohorts	
He et al. 2022	1	1	1	1	1	1	1	1	8
Xu et al. 2024	1	1	1	1	2	1	1	1	9
Zhu et al. 2021	1	1	1	1	2	1	1	1	9
Pan et al. 2021	1	1	1	1	1	1	1	1	8

Supplementary Table S2: Quality judgments of included studies using the Newcastle-Ottawa scale.

Operation Time

Omitting	MD	95%-CI	Heterogeneity (*P*)
He et al. 2022	-9.31	-10.21 to -8.41	0.0%
Xu et al. 2024	-8.14	-11.92 to -4.37	82.4%
Zhu et al. 2021	-7.80	-10.88 to -4.72	80.3%
Pan et al. 2021	-7.97	-11.58 to -4.36	81.0%
Pooled estimate	-8.43	-10.90 to -5.96	73.7%

Radiation Dose

Omitting	MD	95%-CI	Heterogeneity (*P*)
He et al. 2022	-8.51	-13.10 to -3.92	98.0%
Xu et al. 2024	-6.82	-12.47 to -1.17	98.5%
Zhu et al. 2021	-5.45	-8.45 to -2.45	95.8%
Pan et al. 2021	-8.15	-13.36 to -2.95	98.6%
Pooled estimate	-7.23	-11.31 to -3.15	98.1%

Injected Cement Volume

Omitting	MD	95%-CI	Heterogeneity (*P*)
He et al. 2022	-0.67	-1.13 to -0.22	87.0%
Xu et al. 2024	-0.99	-1.87 to -0.11	97.4%
Zhu et al. 2021	-0.92	-1.80 to -0.04	98.0%
Pan et al. 2021	-1.19	-1.76 to -0.61	97.0%
Pooled estimate	-0.94	-1.57 to -0.32	97.0%

Cement Leakage Rate

Omitting	OR	95%-CI	Heterogeneity (*P*)
He et al. 2022	0.57	**0.31 to 1.04**	0.0%
Xu et al. 2024	0.48	0.25 to 0.93	3.7%
Zhu et al. 2021	0.48	0.24 to 0.95	3.7%
Pan et al. 2021	0.42	0.22 to 0.79	0.0%
Pooled estimate	0.49	0.28 to 0.85	0.0%

Visual Analogue Scale

Omitting	MD	95%-CI	Heterogeneity (I^2)
He et al. 2022	-0.12	-0.24 to 0.00	34.7%
Xu et al. 2024	-0.06	-0.19 to 0.07	23.7%
Zhu et al. 2021	-0.09	-0.21 to 0.02	55.9%
Pan et al. 2021	-0.10	-0.23 to 0.03	47.9%
Pooled estimate	-0.09	-0.20 to 0.01	41.0%

Oswestry Disability Index

Omitting	MD	95%-CI	Heterogeneity (I^2)
He et al. 2022	-0.49	-1.80 to 0.82	84.6%
Xu et al. 2024	0.69	-0.05 to 1.43	0.0%
Zhu et al. 2021	-0.17	-1.61 to 1.27	88.2%
Pan et al. 2021	-0.17	-1.70 to 2.17	85.7%
Pooled estimate	-0.03	-1.19 to 1.13	84.4%

Cobb/kyphotic Angle

Omitting	SMD	95%-CI	Heterogeneity (I^2)
He et al. 2022	0.14	-0.13 to 0.41	65.0%
Xu et al. 2024	0.27	**0.03 to 0.51**	52.1%
Zhu et al. 2021	0.26	**0.03 to 0.50**	54.7%
Pan et al. 2021	0.06	-0.11 to 0.24	0.0%
Pooled estimate	0.19	-0.02 to 0.40	53.3%

Supplementary Table S3: Sensitivity analysis table for operation time, radiation dose, injected cement volume, cement leakage rate, VAS, ODI, and Cobb/kyphotic angle. Data in bold indicate pooled results that changed.

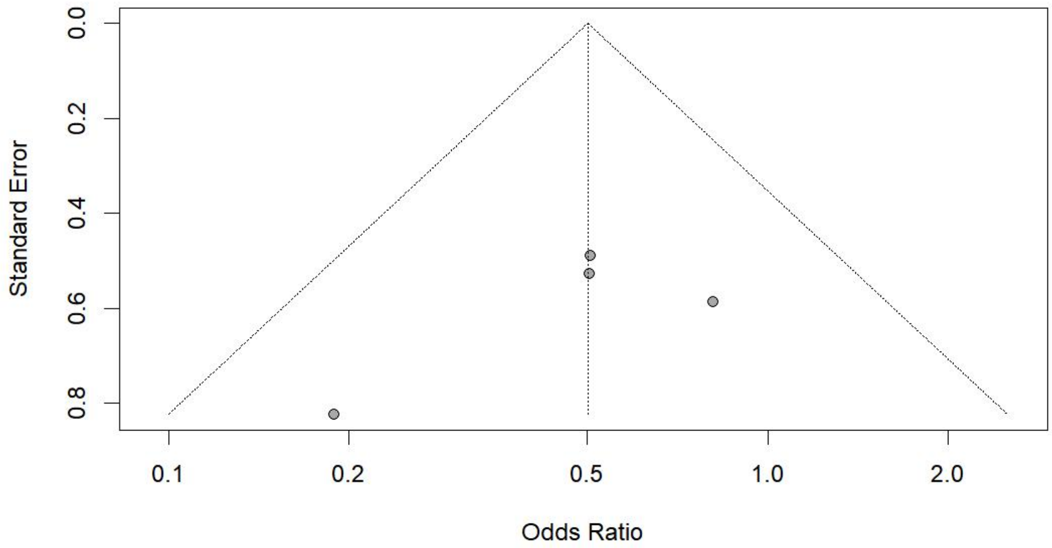

Supplementary Figure S1: A funnel plot of the cement leakage rate.
